# Anticancer and Antioxidant Activity of Bread Enriched with Broccoli Sprouts

**DOI:** 10.1155/2014/608053

**Published:** 2014-06-24

**Authors:** Urszula Gawlik-Dziki, Michał Świeca, Dariusz Dziki, Łukasz Sęczyk, Urszula Złotek, Renata Różyło, Kinga Kaszuba, Damian Ryszawy, Jarosław Czyż

**Affiliations:** ^1^Department of Biochemistry and Food Chemistry, University of Life Sciences, Skromna Street 8, 20-704 Lublin, Poland; ^2^Department of Thermal Technology, University of Life Sciences, Doświadczalna Street 44, 20-280 Lublin, Poland; ^3^Department of Equipment Operation and Maintenance in Food Industry, University of Life Sciences, Doświadczalna Street 44, 20-280 Lublin, Poland; ^4^Department of Cell Biology, Jagiellonian University, Gronostajowa Street 7, 30-387 Cracow, Poland

## Abstract

This study is focused on antioxidant and anticancer capacity of bread enriched with broccoli sprouts (BS) in the light of their potential bioaccessibility and bioavailability. Generally, bread supplementation elevated antioxidant potential of product (both nonenzymatic and enzymatic antioxidant capacities); however, the increase was not correlated with the percent of BS. A replacement up to 2% of BS gives satisfactory overall consumers acceptability and desirable elevation of antioxidant potential. High activity was especially found for extracts obtained after simulated digestion, which allows assuming their protective effect for upper gastrointestinal tract; thus, the anticancer activity against human stomach cancer cells (AGS) was evaluated. A prominent cytostatic response paralleled by the inhibition of AGS motility in the presence of potentially mastication-extractable phytochemicals indicates that phenolic compounds of BS retain their biological activity in bread. Importantly, the efficient phenolics concentration was about 12 *μ*M for buffer extract, 13 *μ*M for extracts after digestion *in vitro*, and 7 *μ*M for extract after absorption *in vitro*. Our data confirm chemopreventive potential of bread enriched with BS and indicate that BS comprise valuable food supplement for stomach cancer chemoprevention.

## 1. Introduction

Stomach cancer, the second most common cancer in the world, represents a very important health problem with about 900,000 new cases diagnosed every year. Despite advances in diagnosis and treatment, the 5-year survival rate of stomach cancer is only 25% [1]. The etiology of stomach cancer is multifactorial and predominantly dietary. Accumulating evidence supports the hypothesis that several medicinal plants and phytochemicals offer chemoprotection against toxic mutagenic and carcinogenic chemicals. There are some reports stating that their anticarcinogenic effects are linked with a high antioxidant capacity (abilities to scavenge reactive oxygen species (ROS) and modulate enzymatic antioxidant defense) [1–3].

The group of secondary plant metabolites with well-documented biological activity is phenolic compounds [4]. Many epidemiological studies proved that consumption of food with high phenolics content is associated with the prevention of many pathological disorders, for example, coronary disease and cancer [5, 6]. It is thought that dietary antioxidants can enhance cellular defense and help to protect cellular components against oxidation damage. Most importantly, the whole group of antioxidants participates in an antioxidative response, not only one of their kinds. Although the biological activity is strongly determined by interactions of antioxidants (synergism, antagonism, and additive effect), there are only a few studies concerning this issue in so copmlicated system which is a whole food. Additionally, the biological properties of antioxidants may depend on their release from the food matrix during the digestion process (bioaccessibility) and may differ quantitatively and qualitatively from those produced by the chemical extraction employed in most studies [7]. Thus, for studying structural changes, digestibility, and release of food components,* in vitro* digestion models are widely used.

One of the most valuable sources of the multidirectional prohealth phytochemicals is broccoli sprouts (BS). Young BS, as a functional food, contain many bioactive, health-promoting compounds. They have been recognized as a rich source of versatile biologically active compounds (such as flavonoids and phenolic acids including gallic, chlorogenic, ferulic, sinapinic, benzoic, and salicylic acids, quercetin, kaempferol, and other endogenous metabolites—vitamin C and glucosinolates) with documented anticancer activity. Protective elements in a cancer prevention diet include selenium, folic acid, vitamin B-12, vitamin D, chlorophyll, and antioxidants. In animal and* in vitro* models, broccoli sprouts phytochemicals show also antihypertensive, anticancer, cardioprotective, and hypocholesterolemic abilities and have bactericidal properties against* Helicobacter pylori*  [8, 9]. Our previous studies clearly show that BS contain compounds able to inhibit the activity of some prooxidant enzymes such as lipoxygenase (LOX) and xanthine oxidase (XO) and to activate antioxidant enzymes such as catalase (CAT) and superoxide dismutase (SOD) [3, 10–12].

There is growing evidence that diets rich in phenols and polyphenols may have potential health benefits for consumers. The best vehicle for functional supplements, due to the widespread consumption (in developed communities they provide more than 50% of the total energy intake), is considered cereals food products (e.g., bread) [13]. Somehow wheat bread possesses some antioxidant capacity; its fortification is justifiable due to deficit of antioxidants in the common diet. So far, there are some successful trials concerning improvement of nutraceutical potential of bread by fortification [14–18]. Thus, we proposed a new functional product—wheat bread enriched with powdered broccoli sprouts (BS).

This study is focused on the changes of the antioxidant capacity of the bread enriched with BS in the light of its potential bioaccessibility and bioavailability. Furthermore, the effect on* in vitro* proliferation and motility of stomach cancer cells differing in metastatic potential were evaluated. Special attention is also placed on relationships between antioxidants and food matrix.

## 2. Materials and Methods

### 2.1. Chemicals

Ferrozine (3-(2-pyridyl)-5,6-bis-(4-phenyl-sulfonic acid)-1,2,4-triazine), ABTS (2,2′-azino-bis(3-ethylbenzthiazoline-6-sulphonic acid)), NBT (nitro blue tetrazolium), DETAPAC (diethylenetriaminepentaacetic acid), *α*-amylase, pancreatin, pepsin, bile extract, Folin-Ciocalteu reagent, linoleic acid, ammonium thiocyanate, and haemoglobin were purchased from Sigma-Aldrich company (Poznan, Poland). All other chemicals were of analytical grade.

### 2.2. Material

Broccoli (*Brassica oleracea* L. var.* italica* cv. Cezar) seeds were purchased from PNOS S.A. in Ozarow Mazowiecki, Poland. Dry seeds were sterilized with 1% (v/v) sodium hypochlorite for 5 min., rinsed with sterile water, and allowed to imbibe water for 6 h at 25°C. Seeds were germinated in sterile Petri dishes covered with filter paper (Whatman Grade number 2) for 6 days at 25°C and in darkness. The germinating seeds were watered with 6 mL of distilled water per day. Broccoli sprouts (BS) were collected, dried, and powdered using a laboratory mill.

### 2.3. Bread Making

The flour used in the formula of control bread was wheat bread flour (600 g), type 750 (average 0.75% ash content, water content 14% wb). The flour was replaced with BS at 1, 2, 3, 4, 5% levels, bread B1–B5, respectively. Besides this, 6 g of instant yeast and 12 g of salt were used for dough preparation. The general quantity of water necessary for the preparation of the dough was established through the marking of water absorption properties in the flour of consistency of 350 Brabender units. The batches of dough were mixed in a spiral mixer for 6 min. After fermentation, the pieces of dough (300 g) were put into the oven heated up to a temperature of 230°C. The baking time was 30 min. After baking, bread was allowed to cool down to room temperature for 24 h. Subsequently, the bread was sliced (slices about 1.5 cm thick). The crust was removed aseptically and kept frozen (at −20°C) until analysis. After thawing, the slices were dried and then manually crumbed, grounded in a mill, and screened through 0.5 mm sieve to obtain bread powder.

### 2.4. Sensory Evaluation

The sensory evaluation was carried out on bread samples (slices about 1.5 cm thick) with the different percentages of broccoli sprouts. Subsequently, the breads were coded with a number and served to consumers. The panel consisted of 27 consumers (24–45 years old), who evaluated bread overall acceptability. The hedonic test was used to determine the degree of overall liking for the breads based on degree of liking or disliking according to a nine-point hedonic scale (1: dislike extremely, 5: neither like nor dislike, 9: like extremely). Plain water was used for mouth rinsing before and after each sample testing [17].

### 2.5. Extracts Preparation

For buffer extracts (BE) preparation, powdered samples of breads (1 g) were extracted for 1 h with 20 mL of PBS buffer (phosphate buffered saline, pH 7.4). The extracts were separated by decantation and the residues were extracted again with 20 mL of PBS buffer. Extracts were combined and stored in darkness at −20°C. For preparation of extracts after simulated digestion (GD), simulated saliva solution was prepared by dissolving 2.38 g Na_2_HPO_4_, 0.19 g KH_2_PO_4_, 8 g NaCl, and 100 mg of mucin in 1 liter of distilled water. The solution was adjusted to pH = 6.75 and *α*-amylase (E.C. 3.2.1.1.) was added to obtain 200 U per mL of enzyme activity. For the gastric digestion, 300 U/mL of pepsin (from porcine stomach mucosa, pepsin A, EC 3.4.23.1) in 0.03 mol/L NaCl, pH = 1.2, was prepared. Further, simulated intestinal juice was prepared by dissolving 0.05 g of pancreatin (activity equivalent 4x USP) and 0.3 g of bile extract in 35 mL 0.1 mol/L NaHCO_3_ [18]. The bread samples were subjected to simulated gastrointestinal digestion as follows: 1 g of powdered sample was homogenized in a stomacher laboratory blender for 1 min to simulate mastication with the presence of 15 mL of simulated salivary fluid, and, subsequently, the samples were shaken for 10 minutes at 37°C. The samples were adjusted to pH = 1.2 using 5 mol/L HCl, and, subsequently, 15 mL of simulated gastric fluid was added. The samples were shaken for 60 min at 37°C. After digestion with the gastric fluid, the samples were adjusted to pH = 6 with 0.1 mol/L of NaHCO_3_ and then 15 mL of a mixture of bile extract and pancreatin was added. The extracts were adjusted to pH = 7 with 1 mol/L NaOH and finally 5 mL of 120 mmol/L NaCl and 5 mL of mmol/L KCl were added to each sample. The prepared samples were submitted for* in vitro* digestion for 120 minutes, at 37°C in the darkness. After that, samples were centrifuged and supernatants were used for further analysis.

Considering that antioxidants absorption takes place mainly at the intestinal digestion stage, the resulting mixture (fluids obtained after* in vitro* digestion) was transferred to the dialysis sacks (D9777-100FT, Sigma-Aldrich), placed in an Erlenmeyer flask containing 50 mL of PBS buffer, and incubated in a rotary shaker (2 times per 2 h, 37°C). The PBS buffer together with the compounds that passed through the membrane (dialysate) was treated as an equivalent of the raw material absorbed in the intestine after digestion (GDA) [18].

### 2.6. Total Phenolics Content

Total phenols were estimated according to the Folin-Ciocalteu method [19]. A 0.1 mL sample of the extract was mixed with 0.1 mL of H_2_O, with 0.4 mL of Folin reagent (1 : 5 H_2_O) and after 3 min with 2 mL of 10% Na_2_CO_3_. After 30 min, the absorbance of mixed samples was measured at a wavelength of 720 nm. The amount of total phenolics was expressed as gallic acid equivalents (GAE).

### 2.7. Antioxidant Capacity

#### 2.7.1. Free Radicals Scavenging Ability (ABTS)

The experiments were performed using an improved ABTS decolorization assay [20]. ABTS^+•^ was generated by the oxidation of ABTS with potassium persulfate. The ABTS radical cation (ABTS^+•^) was produced by reacting 7 mmol/L stock solution of ABTS with 2.45 mmol/L potassium persulphate (final concentration). The ABTS^+•^ solution was diluted (with distilled water) to an absorbance of 0.7 ± 0.05 at 734 nm. Then, 40 *μ*L of samples was added to 1.8 mL of ABTS^+•^ solution and the absorbance was measured at the end time of 5 min. The ability of the extracts to quench the ABTS free radical was determined using the following equation:

(1) $$\begin{array}{c} \text{scavenging}\;\;\%=\left[\frac{\left(A_{C}-A_{A}\right)}{A_{C}}\right]\times 100, \end{array}$$

where *A*_*C*_ is absorbance of control and *A*_*A*_ is absorbance of sample.

Antiradical activity was determined as EC_50_: extract concentration provided 50% of activity based on dose-dependent mode of action.

#### 2.7.2. Metal Chelating Activity

Chelating power was determined by the method of Guo et al. [21]. The extract samples (0.5 mL) were added to a 0.1 mL of 2 mM FeCl_2_ solution and 0.2 mL 5 mM ferrozine and the mixture was shaken vigorously and left standing at room temperature for 10 min. Absorbance of the solution was then measured spectrophotometrically at 562 nm. The percentage of inhibition of ferrozine-Fe^2+^ complex formation was given in the below formula:

(2) $$\begin{array}{c} \%\;\;\text{inhibition}=\left[1-\left(\frac{A_{P}}{A_{C}}\right)\right]\times 100, \end{array}$$

where *A*_*C*_ is absorbance of the control and *A*_*P*_ is absorbance in the presence of the sample.

Metal chelating activity was determined as EC_50_: extract concentration provided 50% of activity based on dose-dependent mode of action.

#### 2.7.3. Ferric Reducing Power (FRAP)

Reducing power was determined using the method described by Oyaizu [22]. Extracts (2.5 mL) were mixed with phosphate buffer (2.5 mL, 200 mmol/L, pH 6.6) and 2.5 mL of 1 g/100 mL aqueous solution of potassium ferricyanide K_3_[Fe(CN_6_)]. The mixture was incubated at 50°C for 20 min. A portion (0.5 mL) of 10 g/100 mL trichloroacetic acid was added to the mixture, which was then centrifuged at 25 ×g for 10 min. The upper layer of solution (2.5 mL) was mixed with distilled water (2.5 mL) and 0.5 mL of 0.1 g/100 mL FeCl_3_, and the absorbance was measured at 700 nm. EC_50_ value (mg/mL) is the effective concentration at which the absorbance was 0.5 for reducing power and was obtained by interpolation from linear regression analysis.

#### 2.7.4. Inhibition of Linoleic Acid Peroxidation (LPO)

The antioxidant activity was determined as the degree of inhibition on the peroxidation of linoleic acid according to Kuo et al. [23] with modification. Ten microliters of sample was mixed with 0.37 mL 5 mmol/L phosphate buffer (pH 7) containing 0.05% Tween 20 and 4 mmol/L linoleic acid and then equilibrated at 37°C for 3 min. The peroxidation of linoleic acid in the above reaction mixture was initiated by adding 20 *μ*L 10 mmol/L FeCl_2_ in water, followed by incubation in a shaking bath at 37°C for 10 min. Reaction was stopped by adding 5 mL 0.6% HCl in ethanol. The hydroxyperoxide formed was assayed according to a ferric thiocyanate method with mixing in order of 0.02 mol/L FeCl_2_ (0.1 mL) and 30% ammonium thiocyanate (0.1 mL). The absorbance of sample (*A*_*s*_) was measured at 480 nm with spectrophotometer (Lambda 40, Perkin-Elmer) for 5 min. The absorbance of the base control (*A*_0_) was obtained without adding haemoglobin to the above reaction mixture; the absorbance of the maximal control (*A*_100_) was obtained with no sample addition to the above mixture. Thus, the antioxidative activity of the sample was calculated as

(3) $$\begin{array}{c} AA\left[\%\right]=\left(1-\frac{\left(A_{s}-A_{0}\right)}{\left(A_{100}-A_{0}\right)}\right)\times 100. \end{array}$$

Antioxidant activity was determined as EC_50_: extract concentration provided 50% of activity based on dose-dependent mode of action.

#### 2.7.5. Inhibition of Lipoxygenase (LOXI)

Lipoxygenase activity was determined spectrophotometrically at a temperature of 25°C by measuring the increase of absorbance at 234 nm over a 2 min period [24]. The reaction mixture contained 2.45 mL 1/15 mol/L phosphate buffer, 0.02 mL of lipoxygenase solution (167 U/mL), and 0.05 mL of inhibitor (vegetable extract) solution. After preincubation of the mixture at 30°C for 10 min, the reaction was initiated by adding 0.08 mL 2.5 mmol/L linoleic acid. One unit of LOX activity was defined as an increase in absorbance of 0.001 per minute at 234 nm.

Antioxidant activity was expressed as EC_50_: extract concentration provided 50% of activity based on dose-dependent mode of action.

#### 2.7.6. Inhibition of Xanthine Oxidase (XOI)

The XOI activities with xanthine as a substrate were measured spectrophotometrically [25], with the following modification: the assay mixture consisted of 0.5 mL of test solution, 1.3 mL of 1/15 mol/L phosphate buffer (pH 7.5), and 0.2 mL of enzyme solution (0.01 U/mL in M/15 phosphate buffer). After preincubation of the mixture at 30°C for 10 min, the reaction was initiated by adding 1.5 mL of 0.15 mmol/L xanthine solution. The assay mixture was incubated at 30°C and the absorbance (295 nm) was measured every minute for 10 min. XO inhibitory activity was expressed as the percentage inhibition of XO in the above assay mixture system and was calculated as follows:

(4) $$\begin{array}{c} \%\;\;\text{inhibition}=\left(1-\frac{\Delta A/\min_{\text{test}}}{{\Delta A\min}_{\text{blank}}}\right)\times 100, \end{array}$$

where Δ*A*/min⁡_test_ is the linear change in absorbance per minute of test material 10 and Δ*A*min⁡_blank_ is the linear change in absorbance per minute of blank.

Antioxidant activity was expressed as EC_50_: extract concentration provided 50% of activity based on dose-dependent mode of action.

#### 2.7.7. Catalase Activity Assay (CAT)

Influence on CAT activity was assayed by the method of Claiborne [26] with some modification. The assay mixture consisted of 1.95 mL phosphate buffer (0.05 mol/L, pH 7.0), 1.0 mL H_2_O_2_ (0.019 mol/L), and 0.05 mL of enzyme solution (60 U/mL). The decomposition of H_2_O_2_ which was determined directly by the extinction at 240 nm per unit time (3 min) was used as a measure of catalase activity. The catalase activity was expressed as *μ*mol of H_2_O_2_ consumed per min (method conditions). For determination of an influence on the catalase activity, enzyme was preincubated with studied extracts.

#### 2.7.8. Superoxide Dismutase Assay (SOD)

Influence on SOD activity was determined using a kinetic mode [27]. 2.7 mL of reagent mixture containing 0.07 mmol/L NBT, 1.1 mmol/L DETAPAC, and 0.17 mmol/L xanthine in 50 mmol/L phosphate buffer (pH 7.8) was mixed with 100 *μ*L of studied sample. The SOD stock solution was prepared daily by addition of 3 mL of 50 mm phosphate buffer (pH 7.8) into the SOD reagent vial containing 5382 activity units (corresponding to 4140 U/mg protein). SOD working solutions were obtained by dilution in 50 mmol/L phosphate buffer (pH 7.8) and are prepared as needed. Zero correction was done before addition of 100 *μ*L of SOD solutions under agitation. The reaction was initiated by adding 100 *μ*L of xanthine oxidase solution under agitation. One minute after addition of xanthine oxidase, the agitation is stopped and the absorbance change at 560 nm was monitored at 25°C, against air for 5 min. The rate of change of absorbance variation Δ*A*560/min⁡ of an uninhibited assay (in absence of SOD) should be between 0.015 and 0.025; if not, the XO concentration is adjusted. For determination of an influence on the SOD activity, enzyme was preincubated with studied extracts.

### 2.8. Nutrients Digestibility

#### 2.8.1. Starch Digestibility* In Vitro*

Total starch (TS) content was determined after dispersion of the starch granules in 2 M KOH (50 mg bread sample, 6 mL KOH) at room temperature (30 min, constant shaking) and hydrolysis of the solubilized starch with 80 *μ*L (1 mg/mL) amyloglucosidase (14 U mg^−1^; EC 3.2.1.3) at 60°C for 45 min [28]. Glucose content was determined by using the standard dinitrosalicylic acid (DNSA) method [29]. Total starch was calculated as glucose × 0.9. The free reducing sugar content of the samples was determined in order to correct the obtained total starch values obtained. The sucrose content of the samples was also determined in order to correct the obtained total starch values.

The resistant (RS) starch content was analyzed on the basis of results obtained after simulated gastrointestinal digestion. After digestion* in vitro*, pellet was dispersed with 2 M KOH, hydrolyzed with amyloglucosidase, and liberated glucose was quantified, as described above, for total starch (TS). Resistant starch (RS) was calculated as glucose × 0.9. The* in vitro* digestibility of starch was evaluated on the basis of total starch content (TS) and resistant starch (RS) determined after digestion* in vitro* [30] as follows:

(5) $$\begin{array}{c} \text{SD}\;\left[\%\right]=100\%-\left(\frac{\text{RS}}{\text{TS}}\times 100\%\right), \end{array}$$

where SD is the* in vitro* digestibility of starch, TS is the total starch content, and RS is the resistant starch content.

#### 2.8.2. Protein Digestibility* In Vitro*

The proteins content was determined with the Bradford method [31], using bovine serum albumin as the standard protein. The* in vitro* protein digestibility was evaluated on the basis of total soluble protein content and the content of protein determined after digestion* in vitro* [32] as follows:

(6) $$\begin{array}{c} \text{PD}\;\left[\%\right]=100\%-\left[\left(\frac{\text{Pr}}{\text{Pt}}\right)\times 100\%\right], \end{array}$$

where PD is* in vitro* digestibility of protein, Pt is total protein content, and Pr is content of proteins after* in vitro* digestion.

### 2.9. Analysis of Proteins-Phenolics Interactions

#### 2.9.1. Sample Preparation

Soluble protein samples (4 mL), from BE and DE, were mixed with 4 mL of cold acetone, incubated at −20°C for 2 h, and pelleted by centrifugation at 14.000 ×g for 20 min. The pellet was resuspended in PBS buffer (1 mL), pH 7.4, and analyzed.

#### 2.9.2. High-Performance Liquid Chromatography

The samples were characterized by SEC-HPLC using a Varian ProStar HPLC System separation module (Varian, Palo Alto, USA) equipped with a column (COSMOSIL 5Diol-20-II Packed Column 7.5 mm ID × 300 mm) and a ProStar DAD detector [32]. The column thermostat was set at 30°C. The amount of 20 *μ*L of each sample solution was loaded on the column, and protein and peptides were eluted using a 20 mM PBS buffer, pH 7.4. The flow rate was 1 mL min^−1^. Ultraviolet detection was performed at a wavelength of 280 nm.

#### 2.9.3. Determination of Free Amino Groups

Changes in the content of free amino groups were determined by the method of [33] with some modifications. Protein extracts (see Section 2.8.1) (1000 *μ*L) were added to a 100 *μ*L 0.1% water solution of TNBS (2,4,6-trinitrobenzenesulfonic acid) and left standing at 50°C, in the dark for 60 min. Next, 1000 *μ*L of HCl (0.1 M) was added and incubated at room temperature for 30 min. Absorbance of the solution was then measured spectrophotometrically at 420 nm. Content of free amino groups was carried out by means of a standard curve for L-leucine.

### 2.10. Anticancer Activity

All the experiments were carried out on human stomach cancer AGS cells [34]. For proliferation assay, trypsinised cells were seeded into 6-well flasks (Nunclon) at an initial density of 7.5∗10^3^ cells/cm^2^. 24 h after seeding, the culture medium (RPMI supplemented with 10% foetal bovine serum, all from Sigma) was exchanged or replaced with the medium containing the extracts (administered from stock solutions to reach the final concentrations 1 and 0.1 *μ*g/mL of culture medium). Then, the cells were cultured for the next 72 h, fixed with 3.7 formaldehyde, and stained with 0.5 *μ*g/mL bis-benzimide for 20 min. Twenty randomly chosen microphotographs of Hoechst-stained nuclei were taken with a computer-assisted data acquisition system (Leica DM IRE2) for each condition to calculate the average number of cells per dish. Cytoskeleton architecture was analysed in formaldehyde-fixed, Triton-solubilized cells, stained with rabbit anti-vinculin IgG (number V9131, Sigma) and counterstained with Alexa 488-conjugated goat anti-rabbit IgG (number A11008, Invitrogen), TRITC-conjugated phalloidin (number 77418, Sigma), and Hoechst 33258. Image acquisition was performed with a Leica DMI6000B microscope (Leica Microsystems, Wetzlar, Germany) equipped with the Total Internal Reflection Fluorescence (TIRF) and Interference Modulation Contrast (IMC) modules.

Cell motility was measured by a time-lapse videomicroscopy. For cell motility assay, the cells were plated into culture flasks (Corning, 25 cm^2^) at initial cell densities chosen to compensate for the inhibitory action of the extracts on cell proliferation (200 to 400 cells/mm^2^). The movement of individual cells was recorded immediately or 72 hours after the extract administration along with the culture medium (RPMI, Sigma; see above) using a computer-assisted data acquisition system Leica DMI6000B, recording time: 4 hours, with 5-minute time intervals at 37°C. Cell trajectories (>50 cells, three independent experiments) were pooled and statistically analyzed. The following parameters were estimated: (i) the total length of cell displacement (TLCD; *μ*m), that is, the distance from the starting point directly to the cell's final position; (ii) the total length of cell movement (TLCM; *μ*m), that is, the total length of cell trajectory (4 hrs).

### 2.11. Statistical Analysis

Experimental data were shown as means ± S.D. for biochemical and means ± SEM for anticancer activity assays. In biochemical analyses, statistical significance was estimated through Tukey's test for the data obtained from three independent samples of each extract in three parallel experiments (*n* = 9). For the estimation of the effect on cell proliferation and motility, one SB and one GD extract were taken based on its representative biochemical content and activity, and the results from three independent experiments (*n* = 3) were subjected to statistical analyses using the paired Student's *t*-test and the nonparametric Mann-Whitney test, respectively (*n* = 3). Unless stated otherwise, the statistical tests were carried out at a significance level of *α* = 0.05. Statistical tests were performed using Statistica 6.0 software (StatSoft, Inc., Tulsa, USA).

## 3. Results and Discussion

The results of hedonic tests on different types of bread are given in Table 1. The color of both crust and crumb of the enriched bread was a little greener than that of the control bread. However, it had little negative influence on bread acceptability. The taste, aroma, and overall acceptability of control bread and bread at substitution levels of 1-2% had the highest linking score. Higher levels of BS addition caused a drastic decrease in the notes for the aroma and taste. For texture characteristics, similar relationship was observed. The sensory characteristics linking results indicated that a partial replacement of wheat flour in bread with up to 2% ground BS powder gives satisfactory overall consumer acceptability. However, bread containing 4% and 5% of BS was almost totally unacceptable, which might be due to excessive amounts of BS compounds which negatively affected the aroma, taste, and texture of product.

Total phenolics content determined with Folin-Ciocalteu reagent is often considered as a marker of antioxidant activity. Taking into account diversity and/or interaction between antioxidants, this is a simplification; however, correlations between antioxidant activity and total phenolics content were well documented [4, 15].

**Figure 1:**
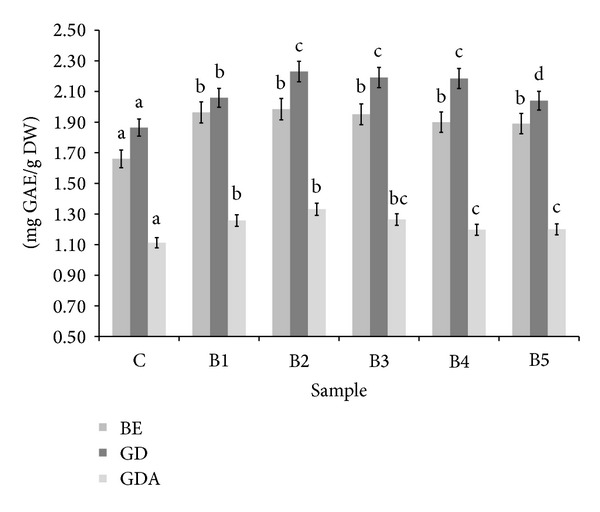
Influence of broccoli sprouts addition on total phenolics content in wheat bread. ^∗^BE: buffer extract, GD: extract after digestion* in vitro*, and GDA: extract after absorption* in vitro*. ^∗∗^Means, within the same kind of extract (BE, GD, and GDA, resp.), with different letters are significantly different (*α* < 0.05); ^∗∗∗^C: control bread, B1–B5: wheat bread with 1–5% of powdered SB addition, respectively.

As being presented in Figure 1, BS addition significantly enriched wheat bread with phenolic compounds; however, there was no linear relationship between the increase of their level and the percent of BS addition. All kinds of bread were rich in buffer-extractable phenolic compounds. In all samples, the highest phenolics content was found after simulated digestion, which may indicate their high bioaccessibility. Potential bioavailability of these compounds was relatively low. Surprisingly, there is a lack of a linear relationship between the BS content and the antioxidant activities of supplemented bread. In most cases, the maximum activity was achieved for the sample B2, and further increase in the share of functional additive does not give the expected results. These results may be partially explained by interactions between food matrix components (especially between phenolics, proteins, and starch) and components of gastrointestinal fluid [32].

**Figure 2:**
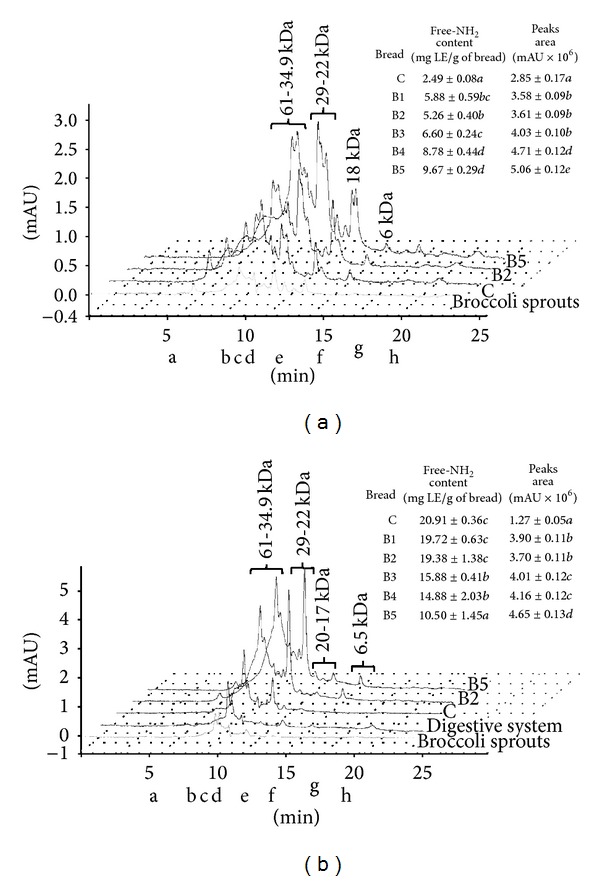
The absorbance profiles of control and enriched bread obtained after size-exclusion chromatography: (a) buffer extracts; (b) extracts after digestion* in vitro*. C: control bread, B2 and B5: bread enriched with 2% and 5% of powdered broccoli sprouts, respectively.  Molecular mass markers (kDa): a: 102; b: 42; c: 35; d: 22; e: 18; f: 6.5; g: 3; h: 1.5. Means, within the same column, with different letters are significantly different (*α* < 0.05).

To determine the protein-phenolics interactions (PPI), SEC techniques were used.  Figure 2(a) showed the absorbance profiles of the buffer extracts of control and enriched bread. The main peaks observed in chromatograms of control bread corresponded with buffer extractable wheat proteins (102-80, 65-35, 30-22, 18, and 6 kDA). What is more, elution profiles obtained for bread fortified with BS are represented by peaks for broccoli sprouts and control bread (Figure 2(a)). Increasing areas of peaks obtained for fortified bread were positively correlated with the percentage addition of BS and also indicate the occurrence of phenolics-protein interactions (Figure 2). In respect to control, the areas of chromatogram obtained for enriched bread were significantly bigger (up to 77% for B5%). Surprisingly, fortification of bread contributed an increase in a level of free amino groups (further studies are needed regarding broccoli sprouts free amino acids content). The chromatographic profiles of extracts obtained after digestion* in vitro* of control and enriched bread showed that the major indigestible protein fractions in control and fortified bread were fractions with molecular weights 61-35, 30-22, 20-16.5, and 6.5 kDa (Figure 2(b)). It should be noted that on chromatograms obtained for digested enriched bread also present were the components of digestive system (DS) and broccoli sprouts (Figure 2(b)) as well as some new peaks characteristic only for fortified bread. Additionally, according to the analysis of chromatograms area it may be stated that supplementation of bread with broccoli sprouts significantly influences protein digestibility. The peaks areas determined for 1%–5% enriched bread were about 3 times higher than those obtained for control bread. A significant decrease of free amino groups in enriched bread was also observed. For B5, their content was lower by about 50% in comparison to control. An increase in peaks area and a reduction in free amino group amounts, starch, and protein digestibility were linked with the percentage addition of BS, what may indicate the presence of interactions between phenolics and proteins from* in vitro* digestive system and/or food matrix proteins (Table 2, Figures 2(a) and 2(b)). The addition of broccoli sprouts to bread affected nutrients digestibility (Table 2). Supplementation of bread with BS increased also the level of resistant starch determined in the basis of simulated digestion. Furthermore, incorporation of BS to bread caused a significant reduction of starch and protein digestibility. Most importantly, these changes were correlated with the percent of functional ingredient. Protein digestibility of B5 was lower by about 60% in respect to control. Protein digestibility of studied bread was inversely proportional to the percentage content of BS. The changes in starch digestibility were not so pronounced as in the case of protein. However, the lowest starch digestibility occurred in bread supplemented with 4% of BS (reduction by 5.5% in respect to control) (Table 2).

The antioxidant properties of food matrices are due to the presence of a complex mixture of compounds of varying polarity. Thus, we decided to use four methods (based on different mechanisms of action) to determine antioxidant capacity of designed products. Taking into account antiradical potential, it can be concluded that BS addition to wheat bread significantly influenced the activity. However, in the buffer extracts (containing potentially mastication-extractable compounds), relatively weak effect was found. Importantly, digestion* in vitro* released antiradical compounds from all enriched bread, when in the control case significant decrease of activity was found (in respect to BE). Antiradical compounds were bioavailable* in vitro*; however, small differences between samples (control and enriched) may indicate higher bioavailability of active compounds derived from the base product (wheat bread) (Table 3). All samples showed also reducing activity. Taking into account the potentially mastication-extractable compounds (BE), the highest activity was found for B2 sample. In other samples, reducing power was significantly lower. Contrary to the assumptions, digestion* in vitro* did not cause any increase in the activity; however, the highest activity was also determined for B2 sample. Moreover, bioavailability of reductive compounds was relatively low. However, contrary to antiradical activity, obtained results indicated bioavailability of reductive compounds derived from functional supplement.

Ability to chelate the metal ions plays an important role in the creation of antioxidant activity. As being presented in Table 2, supplementation of wheat bread with BS caused an increase of this activity in case of hydrophilic compounds (BE); however, no simple linear relationship was observed. In respect to BD, digestion* in vitro* did not increase tested activity. However, it should be mentioned that, for potentially bioaccessible compounds from B1–B3 samples, the activities were significantly higher than those determined for control (wheat bread without BS addition). Most importantly, except the fact that the highest activity was found for control sample, the activity of potentially bioavailable metal chelators was significantly higher than those determined for BE and GE (Table 2). Most importantly, all samples containing potentially mastication-extractable compounds were able to prevent lipids against oxidation. Activity of enriched bread did not depend on the percentage of functional supplement. Surprisingly, digestion* in vitro* did not release active compounds from bread; the activities of enriched bread were significantly lower than those determined for buffer extracts. Beside this, potential bioavailability of lipids preventers was high (Table 2).

**Figure 3:**
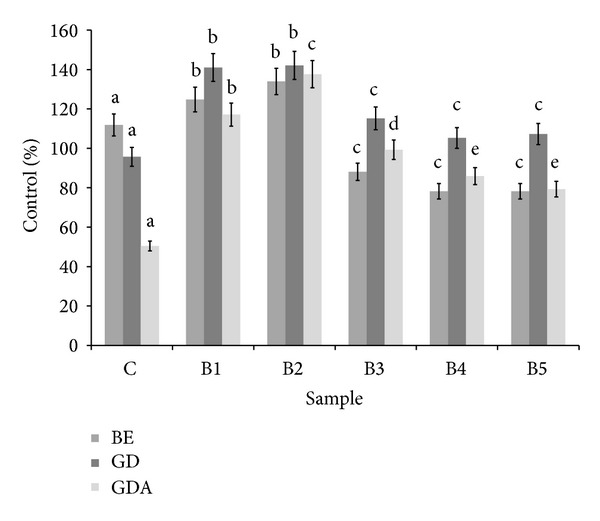
Influence of bread extracts on catalase activity. ^∗^BE: buffer extract, GD: extract after digestion* in vitro*, and GDA: extract after absorption* in vitro*. ^∗∗^Means, within the same kind of extract (BE, GD, and GDA, resp.), with different letters are significantly different (*α* < 0.05); ^∗∗∗^C: control bread, B1–B5: wheat bread with 1–5% of powdered SB addition, respectively.

**Figure 4:**
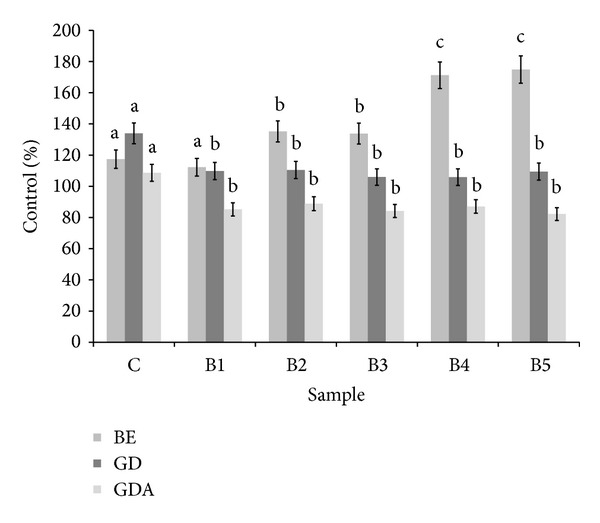
Influence of bread extracts on superoxide dismutase activity. ^∗^BE: buffer extract, GD: extract after digestion* in vitro*, and GDA: extract after absorption* in vitro*. ^∗∗^Means, within the same kind of extract (BE, GD, and GDA, resp.), with different letters are significantly different (*α* < 0.05); ^∗∗∗^C: control bread, B1–B5: wheat bread with 1–5% of powdered SB addition, respectively.

An important part of redox homeostasis includes enzymatic antioxidant system including* inter alia*, CAT, and SOD. Interesting data were obtained by analyzing the effect of bread samples on CAT activity. Taking into account buffer extracts, CAT was activated by samples obtained from control, B1, and B2 bread, wherein the highest activity was found for B2 sample. Interestingly, further increase of BS addition caused a loss of the ability, for B3–B5 samples inhibition of CAT was observed. Most importantly, digestion* in vitro* released CAT activators from all samples except control. The highest activity was found for B1 and B2 bread and CAT activators from theses samples were bioavailable* in vitro* (Figure 3). Buffer extracts of all bread samples were able to activate SOD. Linear relationship between BS addition and ability of SOD activation was found (*R* ^2^ = 0.87). Unexpectedly, only in control bread case, digestion* in vitro* caused an increase of ability to activate SOD (by about 40%), whereas other samples did not affect, significantly, the SOD activity. SOD activators were poorly bioavailable* in vitro*. Surprisingly, B1–B5 samples obtained after simulated absorption exhibited a slight SOD inhibition (by about 12–17%), whereas in the control sample a slight activation (by about 8%) was found (Figure 4).

Superoxide dismutase (SOD) and catalase (CAT) are assumed as biomarkers of chemoprevention owing to their antioxidant and detoxification properties [35]. Kubiak et al. [36] found that patients with colon cancer showed a statistically significant decrease of SOD and CAT activity. More importantly, a significant increase in the level of SOD and lowering CAT activity were observed in all the three categories of breast cancer patients compared to normal individuals [37]. The results suggested that high ROS production supports the oxidative stress in breast cancer. In the light of this, results concerning the changes of SOD and CAT activity (CAT activation and SOD inhibition by potentially bioaccessible B2 samples) may predispose broccoli bread as functional food in secondary therapy.

Content and activity of low-molecular antioxidants and antioxidant enzymes activators may play the crucial role in creating the prohealth properties of plant-derived food; however, equally important is limitation of ROS generation by endogenous factors. Important biological sources of ROS are, among others, LOX and XO. LOXs and their products have also been reported to be important regulators of the proliferation and apoptosis of cancer cell lines; thus, regulation of arachidonic acid metabolism is important in the prevention of many types of cancer, especially cancers of the digestive tracts [38, 39]. XO is considered to be an important biological source of superoxide radicals. These and other reactive oxygen species are involved in many pathological processes such as inflammation, atherosclerosis, and cancer [7, 40].

**Figure 5:**
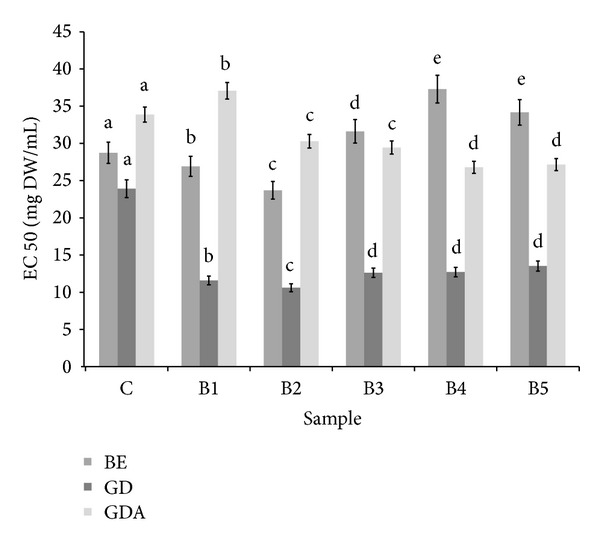
Influence of powdered broccoli sprouts addition on ability to inhibit lipoxygenase activity. ^∗^BE: buffer extract, GD: extract after digestion* in vitro*, and GDA: extract after absorption* in vitro*. ^∗∗^Means, within the same kind of extract (BE, GD, and GDA, resp.), with different letters are significantly different (*α* < 0.05); ^∗∗∗^C: control bread, B1–B5: wheat bread with 1–5% of powdered SB addition, respectively.

**Figure 6:**
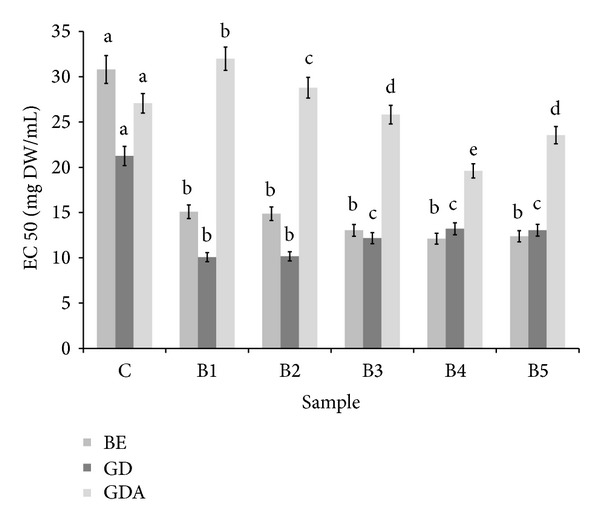
Influence of powdered broccoli sprouts addition on ability to inhibit xanthine oxidase activity. ^∗^BE: buffer extract, GD: extract after digestion* in vitro*, and GDA: extract after absorption* in vitro*. ^∗∗^Means, within the same kind of extract (BE, GD, and GDA, resp.), with different letters are significantly different (*α* < 0.05); ^∗∗∗^C: control bread, B1–B5: wheat bread with 1–5% of powdered SB addition, respectively.

All tested samples possessed ability to inhibit LOX. Taking into account buffer extracts, the highest activity was determined for B2 sample. Particular attention should be paid to the fact that digestion* in vitro* released LOX inhibitors from all tested samples. Activities of enriched bread were significantly higher than those determined for control sample; however, they did not depend on percentage of BS supplementation. Potential bioavailability of LOX inhibitors was relatively low (Figure 5). Buffer-extractable compounds from bread were also able to inhibit XO activity. Addition of BS caused a significant increase of this activity; however, similarly to LOX inhibitors, there was no linear relationship between the level of activity and the percent of BS. Importantly, XO inhibitors were highly bioaccessible* in vitro*; however, activities of all samples were comparable. Unfortunately, XO inhibitors were poorly bioavailable* in vitro* (Figure 6). There is the lack of a linear relationship between the BS content and the antioxidant activity of supplemented bread, which in the light of the results concerning phenolics-bread matrix interactions (Table 3, Figure 2) may confirm a crucial role of phenolics in the creation of antioxidant capacity.

In the light of very promising results obtained for bread with 2% BS supplement, concerning the consumer acceptability, antioxidant activity (antiradical, reducing, chelating and lipids-preventing, activation CAT and SOD), and ability to inhibit the LOX and XO (prooxidative enzymes involved, inter alia, in cancer promotion and progression), further studies of potential anticancer activity were performed. As the especially high activity was found for extracts obtained after simulated digestion a model system stomach cancer was selected.

Frequent attention has recently been directed towards the pleiotropic effects of dietary plant phytochemicals, particularly phenolic compounds, on basic events crucial for cancer initiation, promotion, and progression [41, 42]. In particular, to their activity interference with reactive oxygen species (ROS) has been ascribed. Accordingly, phytochemicals can directly interfere with signaling systems involved in the regulation of inflammatory processes, angiogenesis, and cancer invasion in a manner dependent on their antioxidative activity and concomitant inhibitory effect on the function of protein kinases [41, 43]. However, ROS are also involved in physiological regulation of the signaling pathways, which determine cancer cell proliferation and motility [44].

**Figure 7:**
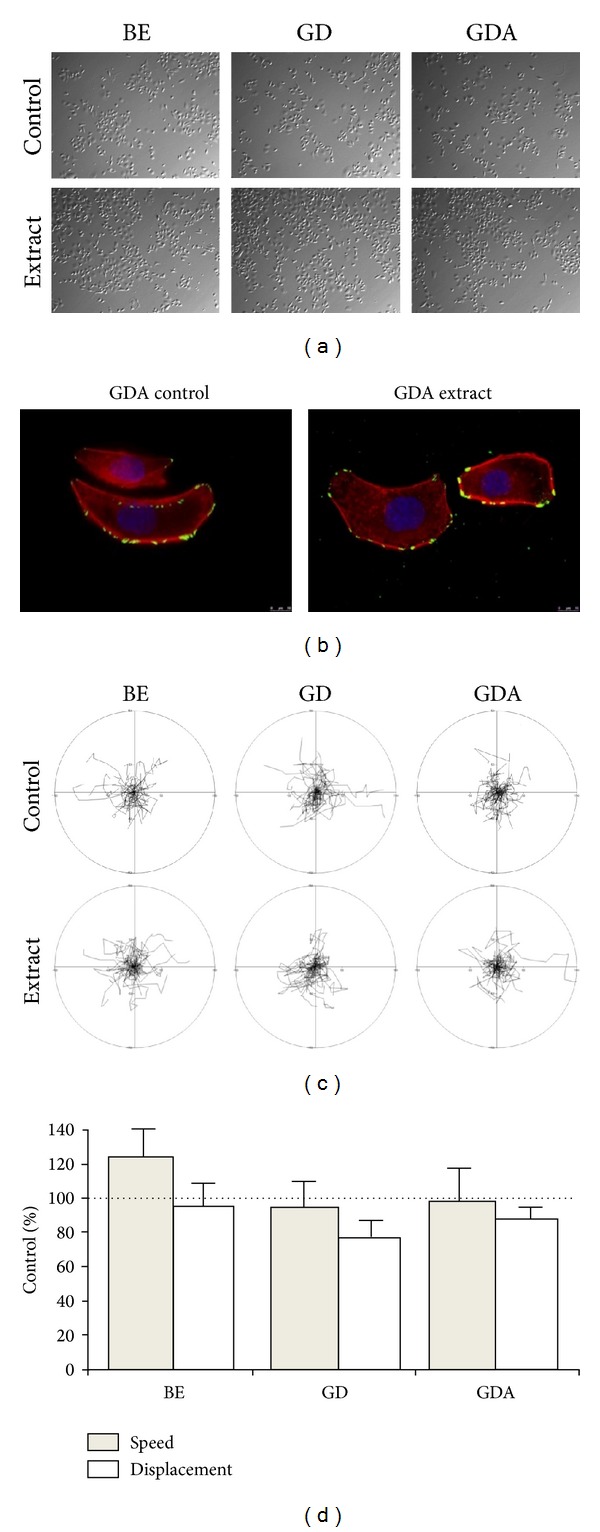
The immediate effect of raw (BE), gastrointestinally digested (GD), and gastrointestinally absorbed (GDA) extracts from breads supplemented with broccoli sprouts on the morphology (a), cytoskeleton architecture (b), and motility ((c), (d)) of AGS cells. The cells cultured in the presence of supplemented extracts administered at the concentration of 1 *μ*g/mL displayed only minute shifts in motility in comparison to their bread controls as demonstrated by circular diagrams (axis scale in *μ*m) drawn with the initial point of each trajectory placed at the origin of the plot (summarised in (d)). Bars represent means ± SEM. ^∗^ *P* < 0.001 determined with the Mann-Whitney test on the data obtained from three independent experiments (*N* = 3).

For the analyses of the antitumorigenic effect of functional product on the motility of cancer cells, BE, GD, and GDA extracts were applied to the cultures of AGS cells at final concentrations of 1 *μ*g/mL. Nonsupplemented (control) bread extracts had no effect on the morphology, proliferation, and motility of AGS cells. Similarly, the extracts from bread supplemented with broccoli sprouts did not exert any significant effects on the morphology (Figure 7(a)) and cytoskeletal architecture (Figure 7(b)) of AGS cells within 8 hours after administration. Accordingly, only a slight increase in the motile activity of AGS was observed in the presence of BE extract, whereas the nonsignificant inhibition of averaged cell displacement was seen in the presence of GD extract (Figures 7(c) and 7(d)).

**Figure 8:**
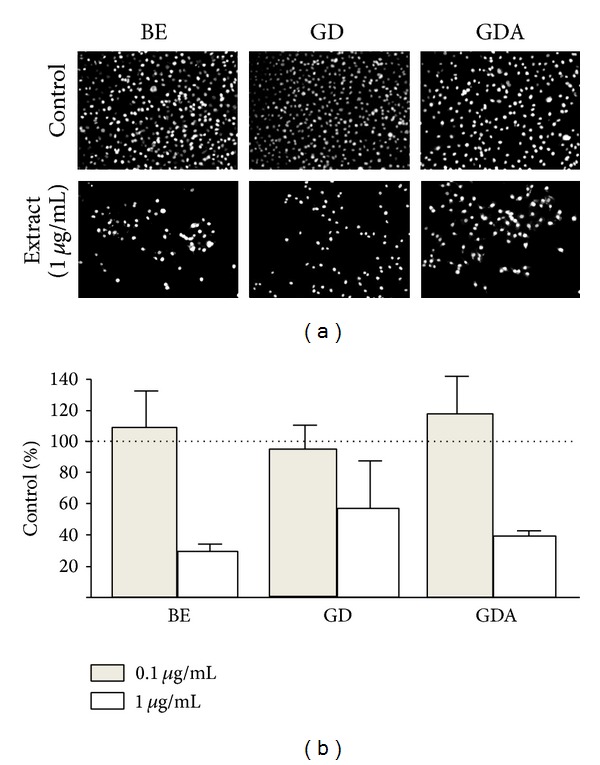
The effect of raw (BE), gastrointestinally digested (GD), and gastrointestinally absorbed (GDA) extracts from breads supplemented with broccoli sprouts on the proliferation of AGS cells. A significant inhibition of AGS proliferation was observed in the presence of all extracts administered at the concentration of 1 *μ*g/mL. No such effect could be seen in the presence of extracts administered at 0.1 *μ*g/mL. SB extract exerted the most pronounced cytostatic effect on AGS proliferation. Bars represent means ± SEM. ^∗^ *P* < 0.05 as determined by the paired Student's *t*-test obtained from three independent experiments.

**Figure 9:**
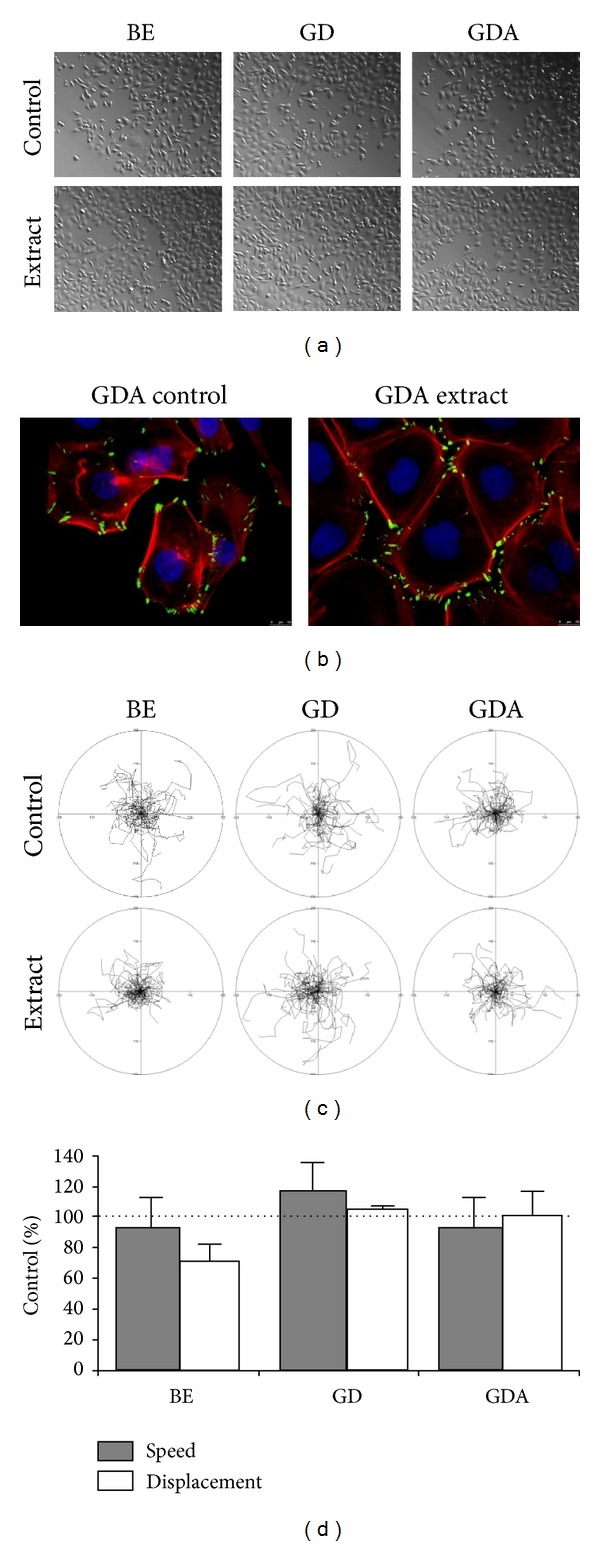
The long-term effect of raw (BE), gastrointestinally digested (GD), and gastrointestinally absorbed (GDA) extracts from breads supplemented with broccoli sprouts on the morphology (a), cytoskeleton architecture (b), and motility ((c), (d)) of AGS cells. The cells cultured in the presence of SB supplemented extract administered at the concentration of 1 *μ*g/mL displayed considerable attenuation of motile activity in comparison to their bread control as demonstrated by circular diagrams (axis scale in *μ*m) drawn with the initial point of each trajectory placed at the origin of the plot (summarised in (d)). Bars represent means ± SEM. ^∗^ *P* < 0.001 determined with the Mann-Whitney test on the data obtained from three independent experiments (*N* = 3).

The biological activity and differences in their phenolic content of BS-supplemented bread extracts were reflected by more pronounced differences in their long-term effects on the proliferation and motility of cancer cells (Figure 8). For instance, 1.0 *μ*g/mL of BE, GD, and GDA extract from supplemented bread reduced AGS proliferation to ca. 30%, 56%, and 45% of its control value. No effect of the extracts administered at the concentration of 0.1 *μ*g/mL could be seen after 72 hours of incubation. The pronounced influence of BE extract on cell proliferation was accompanied by the considerable inhibition of AGS displacement rates (Figures 9(c) and 9(d)) in the absence of any effect on cell morphology and cytoskeleton architecture (Figures 9(a) and 9(b)). Importantly, inhibition of AGS proliferation in the presence of GD and GDA extracts was not accompanied by a long-term attenuation of their motility. These data indicate that phenolic compounds of broccoli sprouts retain their biological activity in bread, but the differences between the activity of BE, GD, and GDA extracts hardly reflect the shifts in their proportions upon gastrointestinal digestion and adsorption. A model based on the analyses of cell proliferation and motility in stomach cancer AGS cell populations* in vitro* enabled the assessment of the importance of anticancerogenic activity of broccoli extracts [45, 46]. These cellular traits, crucial for cancer promotion and progression, respectively, were differentially affected by supplemented extracts [47, 48]. A prominent cytostatic response paralleled by the inhibition of AGS motility in the presence of BE extract indicates that phenolic compounds of BS retain their biological activity in bread. Previously, we have shown that the shifts in the relative content of phenolic compounds in the pure BE and GD extracts failed to correlate with extracts' activity [3]. Our current data remain in agreement with these observations. Regardless of the shifts in their phenolic content, long-term effects of the extracts on cell proliferation and motility of AGS cells are reduced by gastrointestinal digestion and absorption. Importantly, the efficient TPC (estimated as gallic acid equivalent), for the extracts administered at the concentration of 1 mg d.w./mL, was about 12 *μ*M for BE extract, 13 *μ*M for GD, and 7 *μ*M for GDA extract, that is, close to the physiologically relevant values. Altogether, these observations remain in agreement with the findings on the effect of vegetables on the function of cancer cells [41, 43, 49, 50].

## 4. Conclusion

Bread enriched with broccoli sprouts is a valuable source of potentially bioaccessible and bioavailable low-molecular antioxidants and enzyme effectors. In this work, we showed that a partial replacement of wheat flour in bread with up to 2% ground BS powder gives satisfactory overall consumer acceptability. Antioxidants included in functional products exhibit multidirectional activity, which may be translated into high efficiency. Based on the presented data it may be concluded that gastrointestinal digestion and absorption strongly affect potential biological activity. The changes of antioxidant capacity and nutrients digestibility indicate that, in the complex system (such as whole bread), there are interactions strongly limiting the activity of potentially bioactive compounds. The multidirectional biological activity allows assuming their protective effect; thus, the behavior of stomach cancer cells, which may partly be exposed to the compounds unaffected by gastrointestinal processing, was studied. Our data confirm chemopreventive potential of bread enriched with broccoli sprouts and indicate that broccoli sprouts may serve as a valuable food supplement preventing upper gastrointestinal system.

## Figures and Tables

**Table 1:** Sensory evaluation of bread prepared by the substitution of wheat flour with broccoli sprouts powder (BS).

BS addition %	Sensory evaluation				
	Crumb color	Aroma	Texture	Taste	Overall
C^∗∗∗^	8.5^∗^ ± 0.38^a∗∗^	8.8 ± 0.38^a^	7.8 ± 0.24^a^	8.4 ± 0.42^a^	8.4 ± 0.43^a^
B1	8.1 ± 0.26^b^	7.4 ± 0.54^b^	7.2 ± 0.48^ab^	7.2 ± 0.40^ab^	7.5 ± 0.38^b^
B2	8.2 ± 0.34^ab^	5.2 ± 0.29^c^	7.4 ± 0.42^ab^	6.5 ± 0.66^c^	7.1 ± 0.33^b^
B3	8.3 ± 0.42^ab^	3.8 ± 0.61^d^	6.8 ± 0.69^ab^	6.2 ± 0.44^c^	6.3 ± 0.56^c^
B4	7.9 ± 0.42^b^	2.0 ± 0.60^e^	5.6 ± 0.58^c^	4.7 ± 0.58^d^	5.1 ± 0.54^d^
B5	8.1 ± 0.31^ab^	0.8 ± 0.56^f^	3.3 ± 0.37^d^	3.2 ± 0.39^e^	3.9 ± 0.41^e^

*Nine-point hedonic scale of sensory evaluation with 1, 5, and 9 representing extremely dislike, neither like nor dislike, and extremely like, respectively.

**Means with different letter superscript within the same column are significantly different (*α* < 0.05).

***C: control bread, B1–B5: wheat bread with 1–5% of BS addition, respectively.

**Table 2:** Influence of powdered broccoli sprouts addition on nutrients digestibility.

Bread	Starch digestibility [%]	Resistant starch [mg/g of bread]	Protein digestibility [%]
C^∗^	75.40 ± 0.51^b*∗∗*^	159.43 ± 5.29^a^	78.02 ± 3.79^d^
B1	74.13 ± 2.22^ab^	195.22 ± 15.50^b^	54.81 ± 2.35^c^
B2	73.16 ± 1.46^ab^	190.21 ± 8.62^b^	37.47 ± 2.43^b^
B3	72.02 ± 1.44^a^	180.60 ± 26.67^b^	35.61 ± 2.49^b^
B4	71.23 ± 1.42^a^	186.89 ± 16.91^b^	36.05 ± 3.98^ab^
B5	72.96 ± 1.82^a^	182.41 ± 21.41^ab^	29.19 ± 6.83^a^

*C: control bread, B1–B5: bread enriched with 1–5% of powdered broccoli sprouts, respectively.

**Means with different letter superscript within the same column are significantly different (*α* < 0.05).

**Table 3:** Antioxidant activity of bread enriched with powdered broccoli sprouts.

Activity	Bread sample	Buffer extract (BE)	Gastrointestinally digested (GE)	Absorbed (GDA)
Antiradical activity [EC_50_ mg DW/mL]	C∗∗	83.31 ± 3.28^aA∗^	96.29 ± 3.48^aB^	22.99 ± 0.28^aC^
	B1	83.93 ± 2.71^aA^	42.13 ± 2.58^bB^	22.16 ± 0.85^aC^
	B2	76.59 ± 1.98^bA^	39.44 ± 1.25^bB^	22.54 ± 0.87^aC^
	B3	70.49 ± 3.01^cA^	41.64 ± 1.66^bB^	18.86 ± 0.59^bC^
	B4	65.64 ± 2.63^dA^	33.78 ± 1.05^cB^	17.50 ± 0.35^cC^
	B5	72.14 ± 3.21^cbA^	35.34 ± 0.97^cB^	19.40 ± 0.92^bC^

Reducing power [EC_50_ mg DW/mL]	C	35.83 ± 1.65^aA^	69.90 ± 2.63^aB^	81.81 ± 3.58^aC^
	B1	29.85 ± 0.86^bA^	60.80 ± 3.01^beB^	69.76 ± 2.16^bC^
	B2	24.43 ± 0.94^cA^	47.50 ± 2.15^cB^	61.35 ± 2.45^cC^
	B3	50.73 ± 1.29^dA^	61.97 ± 2.85^dB^	59.69 ± 1.85^cB^
	B4	56.98 ± 1.94^eA^	57.85 ± 2.16^eA^	51.95 ± 1.21^dB^
	B5	45.14 ± 1.02^fA^	53.15 ± 1.54^eB^	50.62 ± 2.65^dB^

Chelating power [EC_50_ mg DW/mL]	C	37.70 ± 1.52^aA^	54.74 ± 2.33^aB^	19.43 ± 0.60^aC^
	B1	30.39 ± 0.58^bA^	28.45 ± 0.85^bB^	26.57 ± 0.74^bC^
	B2	27.31 ± 1.33^cA^	37.11 ± 1.02^cB^	21.19 ± 0.58^cC^
	B3	28.08 ± 0.96^cA^	43.21 ± 2.11^dB^	23.84 ± 0.54^dC^
	B4	25.86 ± 0.75^dA^	65.77 ± 2.85^eB^	22.45 ± 0.92^dcC^
	B5	26.83 ± 1.12^dA^	71.82 ± 3.26^fB^	24.18 ± 0.18^aC^

Inhibition of lipids peroxidation [EC_50_ mg DW/mL]	C	28.73 ± 0.89^dA^	27.21 ± 1.12^aA^	17.87 ± 0.45^bB^
	B1	12.69 ± 0.53^aA^	19.47 ± 0.84^bB^	7.13 ± 0.12^cdC^
	B2	11.99 ± 0.35^aA^	19.76 ± 0.58^bB^	8.41 ± 0.35^cC^
	B3	11.91 ± 0.42^aA^	20.24 ± 1.03^cB^	6.81 ± 0.46^deC^
	B4	12.19 ± 0.59^aA^	23.55 ± 1.12^dB^	6.63 ± 0.22^eC^
	B5	12.28 ± 0.45^aA^	22.43 ± 0.82^dB^	8.77 ± 0.31^cC^

*Means within each feature (antioxidant activity) with different small letters (column; the % of supplement) and capital letters (rows; the kind of extract) are significantly different (*α* < 0.05).

**C: control bread, B1–B5: wheat bread with 1–5% of powdered broccoli sprouts addition, respectively.
